# TGF-β2-induced EMT is dampened by inhibition of autophagy and TNF-α treatment

**DOI:** 10.18632/oncotarget.23942

**Published:** 2018-01-04

**Authors:** Subhra Dash, Prasad M. Sarashetti, Balaji Rajashekar, Rajdeep Chowdhury, Sudeshna Mukherjee

**Affiliations:** ^1^ Department of Biological Sciences, Birla Institute of Technology and Science (BITS), Pilani, Pilani Campus, Rajasthan, India; ^2^ Genotypic Technology Pvt. Ltd., Bangalore, India; ^3^ Institute of Computer Science, University of Tartu, Estonia

**Keywords:** autophagy, ROS, EMT, TGF-β2, TNF-α

## Abstract

Hepatocellular carcinoma (HCC) typically develops in a chronic inflammatory setting causal to release of a plethora of growth factors and cytokines. However, the molecular effect of these cytokines on HCC progression is poorly understood. In this study, we exposed HCC cells to TGF-β2 (Transforming Growth Factor-β2), which resulted in a significant elevation of EMT (Epithelial to Mesenchymal Transition) like features. Molecular analysis of EMT markers showed an increase at both RNA and protein levels upon TGF-β2 administration along with up-regulation of TGF-β-induced Smad signaling. Induction of EMT was associated with a simultaneous increase in reactive oxygen species (ROS) and cytostasis of TGF-β2-treated cells. Importantly, quenching of ROS resulted in a significant promotion of TGF-β2-induced EMT. Furthermore, cells treated with TGF-β2 also showed an enhanced autophagic flux. Interestingly, inhibition of autophagy by chloroquine-di-phosphate (CQDP) or siRNA-mediated ablation of ATG5 drastically inhibited TGF-β2-induced EMT. Autophagy inhibition significantly increased ROS levels promoting apoptosis. It was further observed that pro-inflammatory cytokine like, TNF-α (Tumor Necrosis Factor-α) can antagonize TGF-β2-induced response by down-regulating autophagy, increasing ROS levels and thus inhibiting EMT in HCC cells. This inhibitory effect of TNF-α is serum-independent. Transcriptomic analysis through RNA sequencing was further performed which validated that TGF-β2-induced autophagic genes are inhibited by TNF-α treatment suppressing EMT. Our study suggests that autophagy plays a pro-metastatic role facilitating EMT by regulating ROS levels in HCC cells and TNF-α can suppress EMT by inhibiting autophagy. We provide unique mechanistic insights into the role of TGF-β2 in HCC cells, along with appropriate cues to effectively control the disease.

## INTRODUCTION

Autophagy is an evolutionarily conserved lysosome-mediated degradation pathway by which cells self-digest selected cellular macromolecules and maintain homeostasis [1]. Autophagy also facilitates cellular survival under various stresses which may include, nutrient deprivation, growth factor depletion and hypoxia [2]. In recent years, autophagy has emerged as a focus of studies in cancer research, as increasing data indicate that autophagy functions as a central step allowing tumor cells to survive under drug/metabolic stress [3]. In contrary, a host of other studies also describe the role of autophagy in antagonizing cell survival and promoting cell death [4]. We assume that autophagy and cell death phenomenon like, apoptosis in cancer cells, might be linked to each other and occur in a cell type, stimulus and context-dependent manner, which needs to be further explored.

Interestingly, cancer develops in a complex milieu and almost invariably in an inflammatory setting. Under such circumstances, the cellular niche or tumor microenvironment (TME) can contribute substantially to the development, metastasis and therapy resistance of tumors. A plethora of cytokines, in the TME, play a crucial role in shaping tumor progression [5]. Many cytokines are also potent modulators of autophagy suggesting that autophagy-cytokine interaction has evolved as an important mechanism regulating pathogenesis of cancer. While, both archetypal cytokines, like, IFN-γ (Interferon) and TNF-α are known to induce autophagy, the classical cytokines like, IL-4 and IL-13 (Interleukins) are known to suppress it [6]. Additionally, recent studies have also shown that autophagy, apart from being regulated by a host of cytokines, can itself directly control secretion of a number of cytokines. Particularly, a perturbation of regular autophagic pathways has been associated with increased secretion of pro-inflammatory cytokines, like, IL-1α, IL-1β and IL-18 [7]. Activated inflammasome complexes are often engulfed by autophagosomes reducing IL-1β secretion and thus acting as an important homeostatic regulator [8]. Also, autophagy has been reported to positively regulate the transcription and secretion of IL-8 and IL-6 [6]. This establishes a strong connection between autophagy and cytokines and enforces the need to study further details of the interaction to facilitate better understanding of the disease.

In this regard, the cytokine, TGF-β has been shown to induce autophagosome formation and enhanced expression of autophagic markers in multiple cancer types, like, human hepatoma, breast cancer cells, etc. [9]. However, similar to the dual role of autophagy, the role of TGF-β in cancers is controversially discussed. It is proposed to function both as a tumor suppressor and also a tumor promoter in a cellular and context dependent manner. At early stages of oncogenesis, it is known to serve as tumor suppressor, in contrary its role in facilitating EMT and metastasis of tumors in advanced cancers is well acclaimed [10]. Also, a recent study by Jiang *et al.* in 2016 shows that sustained TGF-β treatment in mammary epithelial cells can result in induction of autophagy and reversal of EMT [11]. This endorses the contrasting role played by this cytokine purely in a context dependent fashion. Given that, TGF-β is a multifunctional cytokine with multidirectional role extending from inhibition of growth, induction of apoptosis, triggering of EMT, to senescence, all the more emphasizes the need for further investigating the molecular effects of this cytokine in various cancer cells. Also, whether TGF-β induced autophagy in cancer cells facilitates one or more of the diverse TGF-β-mediated cellular functions remain to be explored.

Another important cytokine, with role in autophagy and other varied cellular processes is tumor necrosis factor α (TNF-α). It is reported to induce autophagy in various cancer cells, like, Ewing sarcoma cells [12], human breast cancer [13] and human T lymphoblastic leukemia cells [14]. However, how TNF-α is connected to autophagy is not fully understood and actually differs across various cell types. TNF-α-induced autophagy has been found to be JNK-dependent in vascular smooth cells, ERK-mediated in human breast cancer cells and reactive oxygen species (ROS)-induced in intestinal epithelial cells [12, 14].

All these extensive links, between cytokines like, TGF-β, TNF-α and autophagy has made this field an attractive area of future research. However, the role of TGF-β and TNF-α in autophagy and possible cross talk between the two cytokines in relation to autophagy needs to be further investigated. Also, whether the activation of autophagy in response to TGF-β enhances cancer cell killing or is a counter stress mechanism is still an open-ended question. Here in, our study shows that TGF-β2 treatment leads to a simultaneous induction of cytostasis and EMT like phenotype in Huh7 cells. The cells undergoing EMT were found to utilize autophagy as a pro-survival strategy, as inhibition of later abrogated EMT-like features. Furthermore, we observed that simultaneous exposure of TNF-α with TGF-β2 antagonize its function and attenuate TGF-β2-induced Smad signaling and EMT. Our study addresses the link between EMT, autophagy and functioning of two important cytokines with respect to their role in autophagy regulation, which can be of potential significance to the understanding of the complex cancerous milieu.

## RESULTS

### TGF-β2 induces Smad-dependent EMT

**Figure 1:**
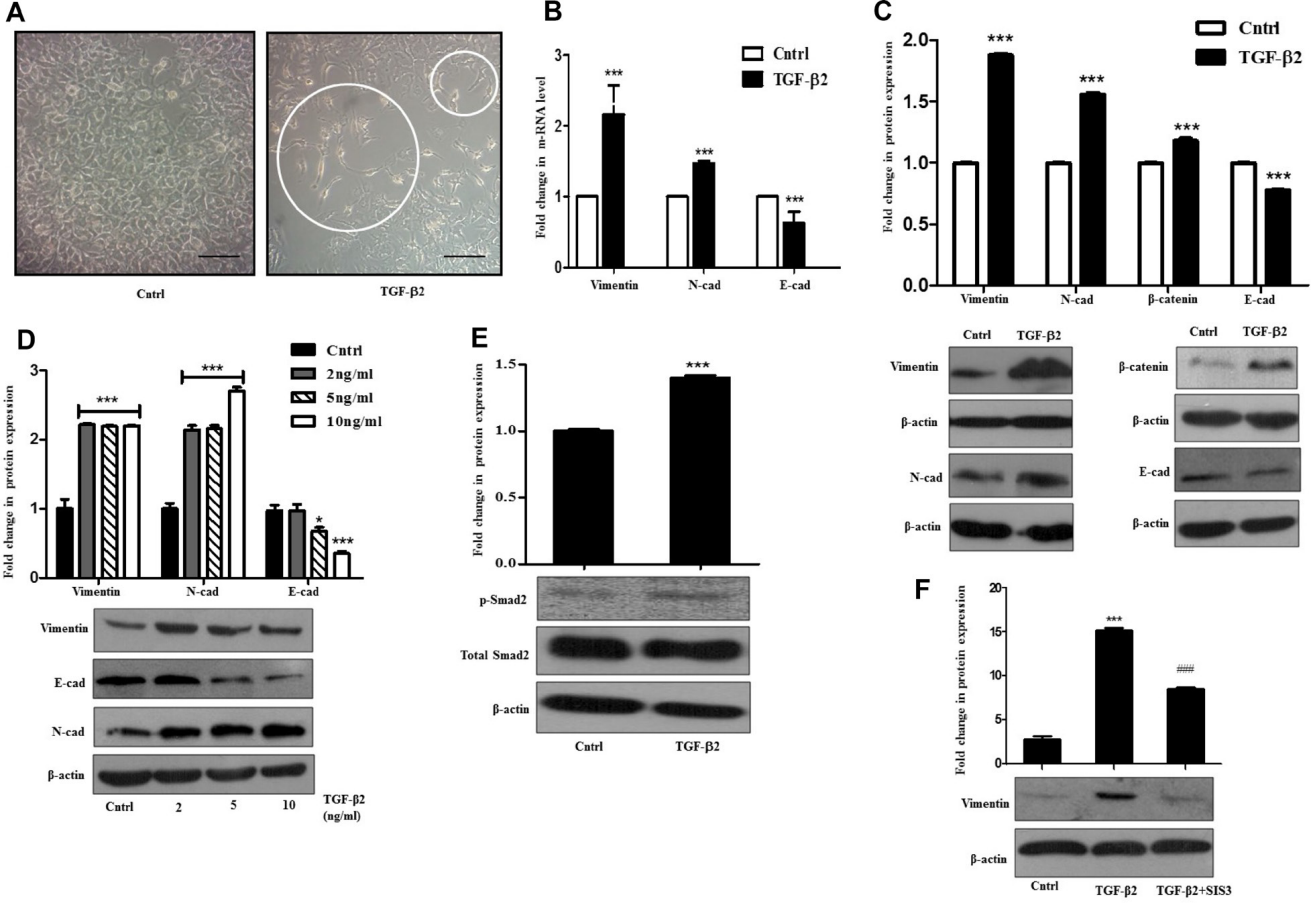
TGF-β2 induces EMT through Smad signaling in Huh7 cells (**A**) Phase-contrast microscopic images of Huh7 cells post 48 h of 5 ng/ml TGF-β2 treatment; white circles indicate elongated cells. The scale bar represents 50 μm, images taken at 40×. (**B**) Quantitative RT-PCR result showing expression of Vimentin, N-cadherin and E-cadherin after 48 h of TGF-β2 (5 ng/ml) treatment. (**C**) Immunoblot assay depicting expression levels of proteins like, Vimentin, N-cadherin, β-catenin and E-cadherin with and without addition of TGF-β2 (5 ng/ml). (**D**) Immunoblot assay showing protein expression of Vimentin, N-cadherin and E-cadherin post TGF-β2 exposure for 48 h at varied concentrations (2, 5, 10 ng/ml). (**E**) Immunoblot assay showing an up-regulation of p-Smad-2 after TGF-β2 treatment (5 ng/ml) for 48 h. (**F**) Immunoblot assay showing protein expression of Vimentin post p-Smad-2 inhibition with SIS3 in TGF-β2 (5 ng/ml; 48 h) treated cells. SIS3 was added 1 h before cytokine treatment. Expression in untreated control was taken as arbitrary unit “1”. β-actin served as a loading control.

TGF-β is a multi-functional cytokine that is known to be involved in tumor suppression, cancer invasion and also for its pro-fibrogenic role in almost all fibrotic diseases [15]. It can effectively orchestrate diverse cellular effects depending on the cell type and context. One of the primary established functions of TGF-β is to promote EMT of cancer cells [16]. EMT can be described as the process promoting metastasis where epithelial cells undergo trans-differentiation by shedding off their polarity and epithelial characteristics, which facilitate their migration into neighboring tissues; and TGF-β is a well-known inducer of it. Taking this into consideration we were interested in exploring TGF-β2-mediated EMT induction in HCC cell type (Huh7) and the signaling associated with it. A distinct change in morphology, marked by extended cellular phenotype was observed in cells exposed to TGF-β2, when compared to untreated control (Figure 1A). Huh7 cells treated separately with an unrelated cytokine e.g., IL-6 in this case, for a similar time period failed to show any change in morphology (Supplementary Figure 1A). During EMT, the down-regulation of E-cadherin is stabilized by the up-regulation of N-cadherin, which connects to the cytoskeleton through β-catenin resulting in a cadherin switch that controls cell adhesion [17]. Another crucial marker for EMT is Vimentin which facilitates invasion of cells into surrounding tissues [18]. In our study, the expression of these key genes was monitored post TGF-β2 exposure through real time PCR and immunoblot. A significant increase in transcript level of N-cadherin and Vimentin was observed along with a concurrent reduction of E-cadherin mRNA (Figure 1B). Immunoblot analysis further revealed a significant increase in Vimentin, N-cadherin and also β-catenin protein levels and a simultaneous decrease in E-cadherin protein levels in Huh7 cells upon 48 h of exposure to TGF-β2 (Figure 1C). Additionally, dose kinetics studies were also performed which showed similar trend in increased expression of N-cadherin and Vimentin at different doses of TGF-β2 with respect to untreated control; similarly, E-cadherin showed a significant decrease in protein levels at higher doses of TGF-β2 (Figure 1D). The altered regulation of the above markers signified an EMT like transition in the epithelial Huh7 cells after TGF-β2 administration. Investigation of the upstream signaling leading to EMT revealed that the canonical TGF-β signaling pathway was activated following TGF-β2 exposure. A high level of phospho-Smad-2 protein was observed in cells undergoing EMT (Figure 1E). To further validate a Smad-dependent effect, TGF-β-induced Smad signaling was inhibited with SIS3, an inhibitor of TGF-β and Activin signaling that acts through specific suppression of Smads without affecting the MAPK/p38, ERK or PI3-kinase signaling pathways [19]. A reduction in Smad levels was observed upon treatment with SIS3, as analyzed by immunoblot analysis (Supplementary Figure 1B). Importantly, a significant reduction in Vimentin protein levels was observed upon Smad inhibition suggesting the role of Smad signaling in TGF-β2-induced EMT (Figure 1F). Taken together, these observations suggest that TGF-β2 treatment induces EMT by activating Smad signaling pathway in Huh7 cells.

### TGF-β2 induced EMT is dependent on ROS levels

**Figure 2:**
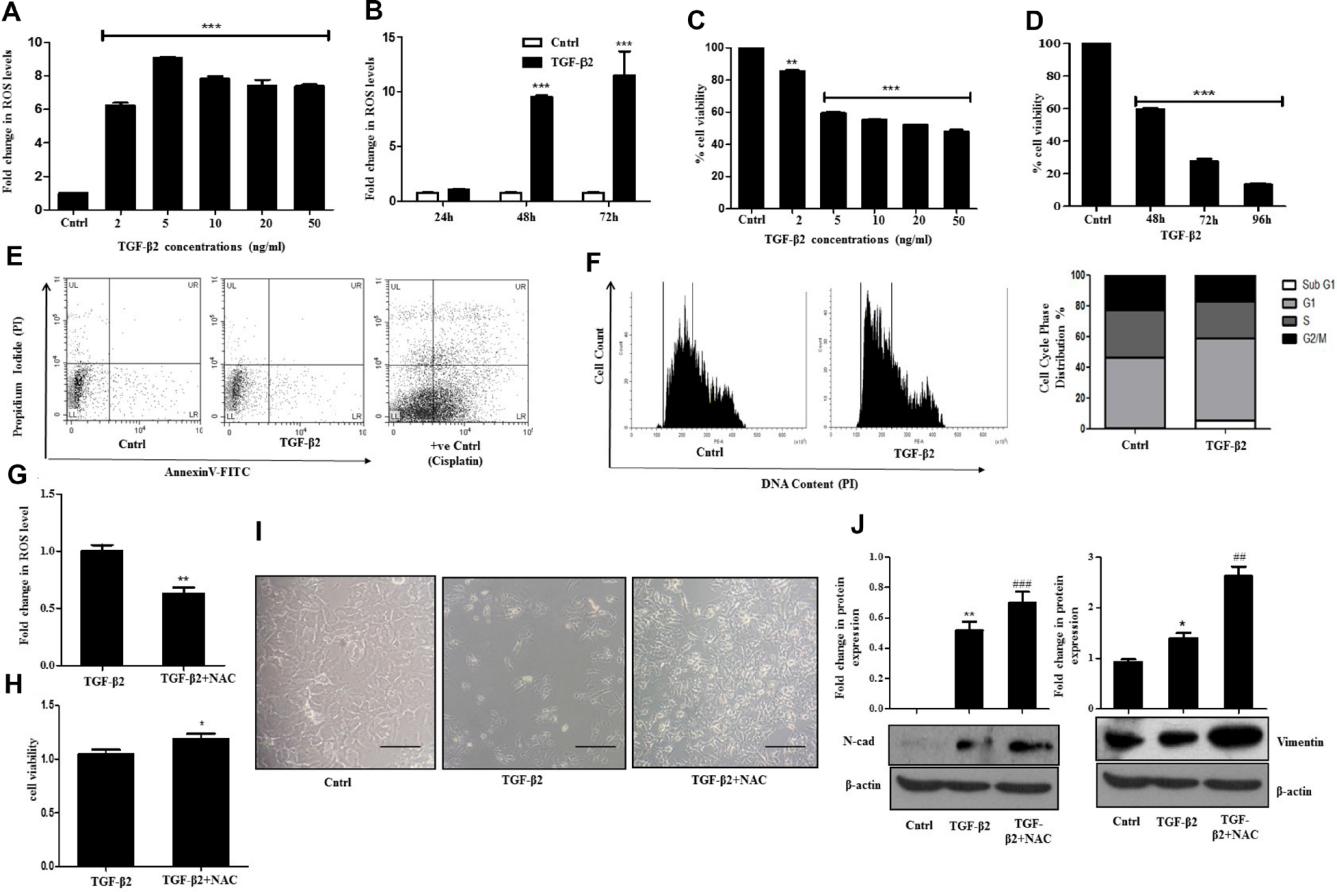
TGF-β2 induced EMT is ROS dependent (**A**) H2DCF-DA fluorimetric assay showing intra-cellular accumulation of ROS after treatment of Huh7 cells with TGF-β2 at the following concentrations (2, 5, 10, 20, 50 ng/ml; 48 h). Untreated control was taken as arbitrary unit “1”. (**B**) Bar graph showing time dependent increase in intracellular ROS accumulation in Huh7 cells after treatment with TGF-β2 (5 ng/ml; 48 h). (**C**) Viability of Huh7 cells measured through MTT assay upon exposure to varied doses of TGF-β2 for 48 h. (**D**) Time dependent alterations in cell viability as analyzed through MTT assay upon treatment with TGF-β2 (5 ng/ml). (**E**) Detection of apoptotic cells after TGF-β2-treatment (5 ng/ml) for 48 h, as analyzed through flow cytometry using AnnexinV-FITC/Propidium Iodide (PI) staining. Cisplatin (35 μm, 24 h) treated cells were taken as positive control. (**F**) Cell cycle analysis of TGF-β2-exposed cells (48 h) using PI dye by flow cytometry. A shift in G1 peak is marked by an arrow. Percentage of cells in each phase of cell cycle is represented by bar diagram. (**G**) Intracellular levels of ROS upon addition of NAC in TGF-β2 (5 ng/ml) exposed Huh7 cells as analyzed through H2DCF-DA fluorimetric assay. (**H**) MTT assay showing percentage cell viability upon inhibition of ROS (5 ng/ml) for 48 h; 5 mM of NAC was added 1 h prior to TGF-β2 exposure (5 ng/ml; 48 h). (**I**) Phase-contrast microscopic images following TGF-β2 (5 ng/ml, 48 h) or TGF-β2 plus NAC treatment for 48 h in Huh7 cells. The scale bar represents 50 μm, images were taken at 40× (**J**) Immunoblot analysis showing expression of N-cadherin and Vimentin upon quenching of ROS with NAC treatment.

Reactive oxygen species (ROS)-induced signaling has often been linked to the diverse activities of TGF-β [20, 21]. TGF-β-induced signaling can lead to redox unevenness through mitochondrial damage or by inhibiting cellular anti-oxidant activities. Recent evidences indicate that TGF-β and ROS can have opposing roles in pro- as well as anti-tumor effects [22, 23]. For these reasons, understanding the interplay between them is important for elucidating TGF-β–mediated effects in cancer progression. In this study, we observed that exposure of Huh7 cells to different doses of TGF-β2 led to a significant increase in intra-cellular ROS levels when compared to un-treated control as analyzed through H2DCF-DA fluorimetric assay (Figure 2A). Time kinetics analysis further showed significantly elevated levels of ROS after 48 h and 72 h of TGF-β2 (5 ng/ml) treatment (Figure 2B). Interestingly, an increase in ROS was associated with TGF-β2-induced cytostatic effect. As compared to untreated control, significantly less number of viable cells was observed upon treatment with different doses of TGF-β2 for varied time points (Figure 2C and 2D). However, treatment with an unrelated cytokine, IL-6 failed to show any cytostatic effect on Huh7 cells (Supplementary Figure 2). To further verify that the reduced viable cell number is not due to cytotoxicity induced by TGF-β2, we performed apoptosis assay with AnnexinV/PI. TGF-β is reported to induce apoptosis in various cell types like, NIH3T3 and AML12 [24]. In our study, at the stipulated dose and time (5 ng/ml; 48 h), TGF-β2 failed to show an induction of apoptosis (Figure 2E). The conventionally used anti-cancer drug, cisplatin (35 µM) was taken as a positive control. To further validate the cytostatic effect, cell cycle analysis was also performed using Propidium Iodide (PI) dye through flow cytometry after TGF-β2 exposure. A significant shift in number of cells in G1 phase of the cell cycle, indicative of arrest at G1/S juncture was observed establishing the cytostatic effect of TGF-β2 in the hepatocellular carcinoma cells studied (Figure 2F). An inhibition of ROS, with the widely used ROS quencher, NAC (N-acetyl cysteine) caused attenuation in ROS levels (Figure 2G) and significant reversal of cytostatic effect induced by TGF-β2 as analyzed by MTT assay (Figure 2H). An increased number of cells was also observed when Huh7 cells were exposed to both NAC and TGF-β2 when compared to only TGF-β2 treatment suggesting a rescue of the cytostatic effect upon quenching of TGF-β2-induced ROS (Figure 2I). This tempted us to investigate the role of ROS in TGF-β2-induced EMT. Interestingly a significant increase in EMT markers like N-cadherin and Vimentin was observed upon NAC-mediated quenching of TGF-β2-induced ROS (Figure 2J). These results suggest that generation of ROS was acting as a limiting factor attenuating EMT induction in Huh7 cells upon exposure to TGF-β2.

### TGF-β2-induced autophagy limits intra-cellular ROS levels

**Figure 3:**
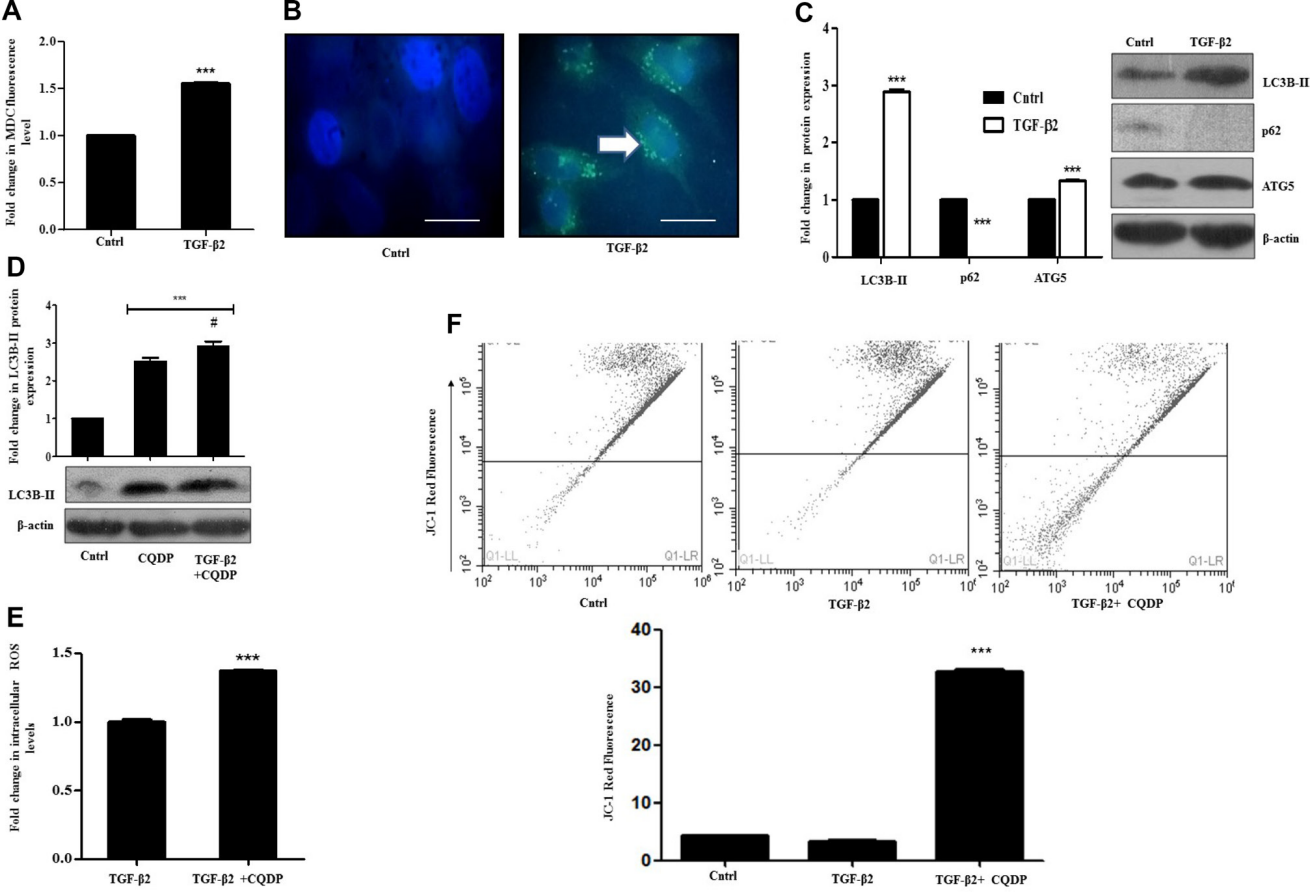
TGF-β2 exposure induces pro-survival autophagy in Huh7 cells (**A**) MDC fluorimetric assay showing autophagosome accumulation in Huh7 cells post 48 h of TGF-β2 treatment (5 ng/ml). (**B**) Fluorescent microscopy images of MDC fluorescence accumulation in TGF-β2-treated (5 ng/ml, 48 h) Huh7 cells. The scale bar represents 50 μm, images were taken at 100×. (**C**) Immunoblot analysis showing expression of autophagic markers- LC3-BII, ATG5 and p62 in TGF-β2-treated (5 ng/ml, 48 h) cells. Expression in untreated control was taken as arbitrary unit “1”. β-actin served as loading control. (**D**) Determination of autophagic flux through immunoblot analysis of LC3B-II accumulation after TGF-β2 exposure (5 ng/ml, 48 h), in presence or absence of CQDP (10 µM); the autophagy inhibitor was added 1 h before addition of TGF-β2. The expression level of TGF-β2 was taken as arbitrary unit “1”. (**E**) H2DCF-DA fluorimetric assay showing fold increase in ROS levels, upon inhibition of autophagy by CQDP (10 µM) in TGF-β2-treated cells (5 ng/ml, 48 h). ROS level in only TGF-β2 treated cells was taken as arbitrary unit “1”. (**F**) JC-1 fluorescence estimation by flow cytometry, showing a decrease in red fluorescence indicative of reduction of mitochondrial membrane potential after exposure to TGF-β2 (5 ng/ml, 48 h) and CQDP (10 µM; added 1 h before cytokine addition), compared to only TGF-β2

TGF-β has been previously reported to induce autophagy in various mammalian cancer cell types, extending from normal bovine mammary epithelial cells to mammary carcinoma cells *in vitro* [25]. Autophagy triggered, in turn can play a dual and paradoxical role either in tumor progression or suppression. It is known to mediate a cytoprotective phenomenon under nutrient deprivation [26], while, there are other evidences which document its role in promoting cell death [27]. The above observations prompted us to investigate whether autophagy is activated by TGF-β2 administration in Huh7 cells, and if so, what role it plays in such context. Exposure of Huh7 cells to TGF-β2 led to an increase in intracellular monodansylcadaverine (MDC) fluorescence indicative of autophagosome formation which was measured fluorometrically (Figure 3A). Microscopic analysis further revealed an increase in the number of MDC labelled spherical autophagic vacuoles distributed in the cellular cytoplasm, when compared to untreated control (Figure 3B). A significant increase in specific autophagic markers like, microtubule-associated protein light chain 3B-II (LC3B-II) and ATG5, indicative of enhanced autophagic activity was also observed by immunoblot analysis upon exposure to TGF-β2 (5 ng/ml; 48 h) (Figure 3C). A lower dose of TGF-β2 (2 ng/ml) for a similar time period also stimulated LC3B-II accumulation indicative of enhanced autophagy (Supplementary Figure 3). Simultaneously, a decrease in p62, the turnover of which serves as a useful marker for induction of autophagy was obtained following exposure of Huh7 cells to TGF-β2 (Figure 3C). Since, the accumulation of autophagosomes is not always indicative of autophagy induction and may just represent either the increased generation of autophagosomes and/or a block in autophagosomal maturation, we hence checked for the difference in the amount of LC3B-II in the presence or absence of the lysosomal inhibitor, chloroquine di-phosphate (CQDP). The difference in the amount of LC3B-II in the presence and absence of CQDP represents the amount of LC3 that is delivered to lysosomes for degradation (i.e., autophagic flux) [23]. An increase in LC3B-II levels, indicative of autophagic flux, was observed in cells treated with CQDP plus TGF-β2 when compared to only CQDP-treated cells (Figure 3D). Considering the induction of autophagy and the context-dependent growth-inhibitory effect of TGF-β2, we were interested to investigate the role of autophagy. Interestingly, the inhibition of autophagy with CQDP caused a significant increase in intracellular ROS levels and also an associated shift in fluorescence of JC-1 dye indicative of a significant dip in mitochondrial membrane potential (Figure 3E and 3F). This was an important observation, given that we previously observed that ROS limits TGF-β2-induced EMT; however how, inhibition of autophagy and hence increased ROS effects EMT remained to be addressed.

### TGF-β2-induced autophagy facilitates EMT by regulating ROS levels

**Figure 4:**
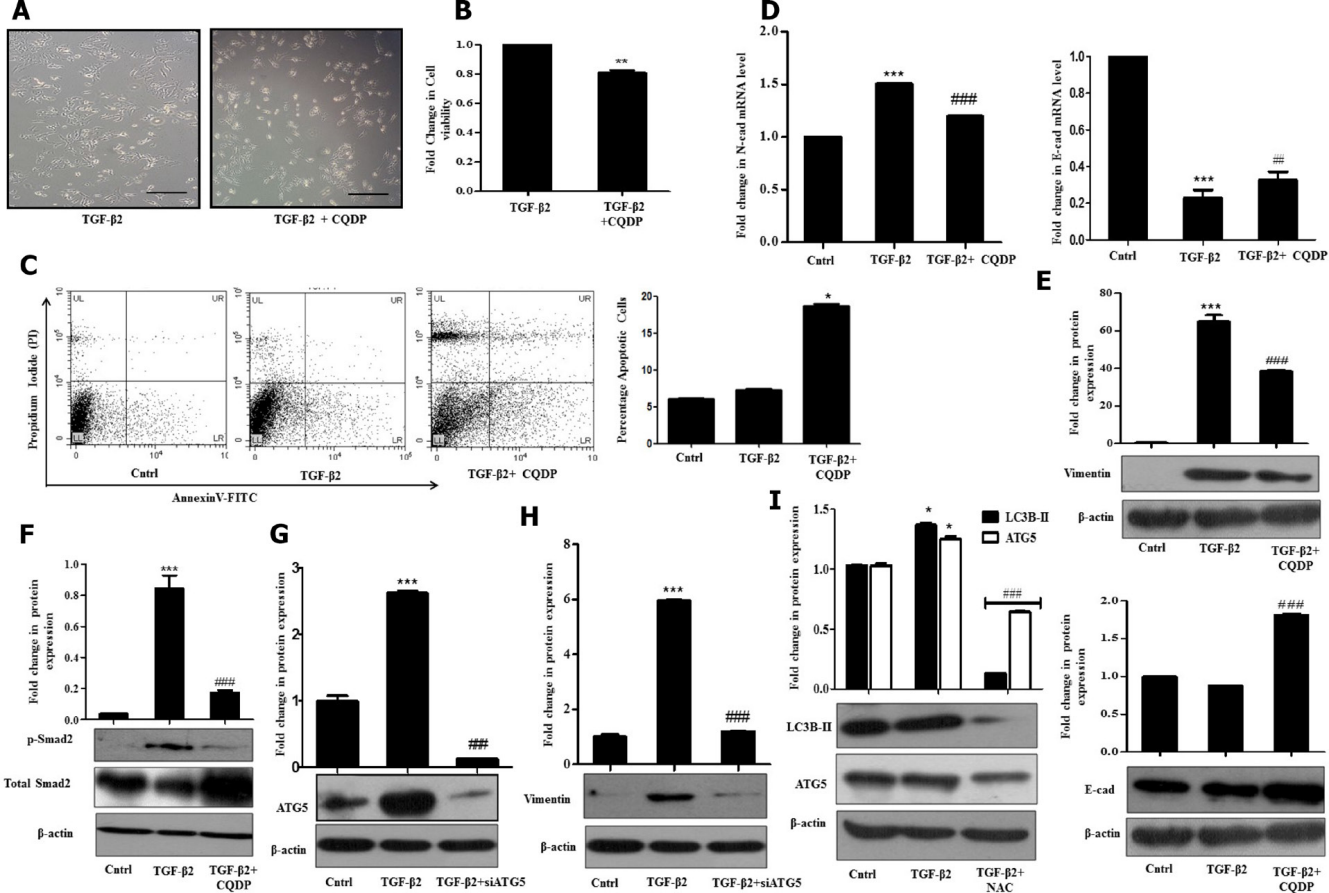
TGF-β2-induced autophagy suppresses ROS and promotes EMT (**A**) Phase contrast microscopic images showing increase in cell death upon inhibition of TGF-β2 (5 ng/ml, 48 h)-induced autophagy with CQDP (10 µM) addition. The scale bar represents 50 μm, images were taken at 40×. (**B**) MTT assay showing percentage cell viability upon inhibition of TGF-β2-induced autophagy by CQDP addition prior to cytokine treatment. The percentage viability of cells after TGF-β2 treatment was taken as arbitrary unit “1”. (**C**) Detection of apoptotic cells following addition of CQDP (10 µM), 1 h prior to TGF-β2 -treatment (5 ng/ml, 48 h) as analyzed through flow cytometry using AnnexinV-FITC/Propidium Iodide (PI) staining. Percentage apoptotic cells are plotted in bar diagram. (**D**) Real time PCR analysis showing expression of N-cadherin and E-cadherin upon autophagy inhibition by CQDP (10 µM) in TGF-β2-treated cells. (**E**) Immunoblot analysis showing Vimentin and E-cadherin protein expression upon autophagy inhibition by CQDP (10 µM) in TGF-β2-treated cells. (**F**) Immunoblot analysis showing p-Smad-2 protein expression upon inhibition of autophagy by CQDP (10 µM) in TGF-β2-treated cells. (**G**) Immunoblot analysis showing knockdown of ATG5 in Huh7 cells transiently transfected with siATG5. (**H**) Immunoblot analysis showing protein expression of Vimentin upon inhibition of autophagy using siATG5. (**I**) Immunoblot analysis showing protein expression of autophagic markers- LC3-BII and ATG5 in untreated control, TGF-β2-treated (5 ng/ml, 48 h) and TGF-β2 plus NAC (5 mM) treated cells. Expression in untreated control was taken as arbitrary unit “1”. GAPDH expression was used as housekeeping control for RT-PCR and β-actin served as loading control for immunoblot.

Cancer cells are known to generate moderate to high levels of ROS to aid their need for enhanced proliferation, migration and metastasis; however, resistance to chemotherapeutic drugs can also be attributed to the higher intra-cellular levels of ROS in cancer cells. In contrary, increasing the level of ROS has often been utilized as a strategy to tip the balance of cancer cells from proliferation towards cell death. Under the above circumstances, the generation of enhanced intracellular ROS plays a pivotal role in initiation of cell death. In corroboration to above, in our study, inhibition of autophagy with CQDP resulted in an increased ROS and an associated cell death of Huh7 cells. Phase contrast images and cell viability analyzed through MTT assay provided evidences for the same (Figure 4A and 4B). To further validate the induction of cell death by autophagy inhibition, apoptosis was analyzed through AnnexinV/PI staining (Figure 4C). A significantly increased cell death was observed in cells treated with TGF-β2 and CQDP, compared to only TGF-β2. At 10 µM concentration, for the studied time period, only CQDP treatment did not show a significant cell death, when analyzed through MTT assay (Supplementary Figure 4). We speculate that TGF-β2-induced autophagy restricts intra-cellular ROS levels to go up substantially probably by clearing off damaged mitochondria; hence, an inhibition of lysosomal activity by CQDP results in a significant boost in intra-cellular ROS levels triggering cell death. However further studies are required to confirm the hypothesis of mitophagy.

Interestingly, inhibition of autophagy with CQDP resulted in a significant decrease in EMT markers, as verified both at the RNA and protein levels (Figure 4D and 4E). Protein levels of Vimentin were significantly reduced upon inhibition of autophagic flux by CQDP (Figure 4E). Furthermore, p-Smad2 levels were found to be significantly going down post CQDP treatment in TGF-β2 exposed Huh7 cells (Figure 4F). To further confirm that inhibition of autophagy has an effect on EMT, we transiently transfected Huh7 cells with siRNA against ATG5. In corroboration to above, knock down of ATG5 (Figure 4G) significantly reduced TGF-β2-induced Vimentin expression level (Figure 4H). The above results are conclusive of the fact that TGF-β2-induced autophagy facilitates EMT by suppressing and thus regulating intra-cellular ROS levels; with inhibition of autophagy ROS levels shoot up suppressing EMT as well as survival of Huh7 cells. To understand, what happens to autophagy levels if ROS is quenched, we checked for autophagic markers post NAC treatment. Interestingly, LC3B-II protein levels significantly went down upon addition of the ROS quencher (Figure 4I). This suggests that autophagy was instrumental in limiting ROS levels, and when intra-cellular ROS was quenched, autophagy was dispensable for EMT, as demonstrated in Figure 2J. Although the role of TGF-β–induced autophagy remains unclear in majority of cell types, and might be different in certain stages and aspects of tumor development, here we establish that autophagy supports TGF-β2–mediated cell survival and induction of EMT in Huh7 cells by controlling intra-cellular ROS.

### TGF-β2–induced EMT is antagonized by TNF-α

**Figure 5:**
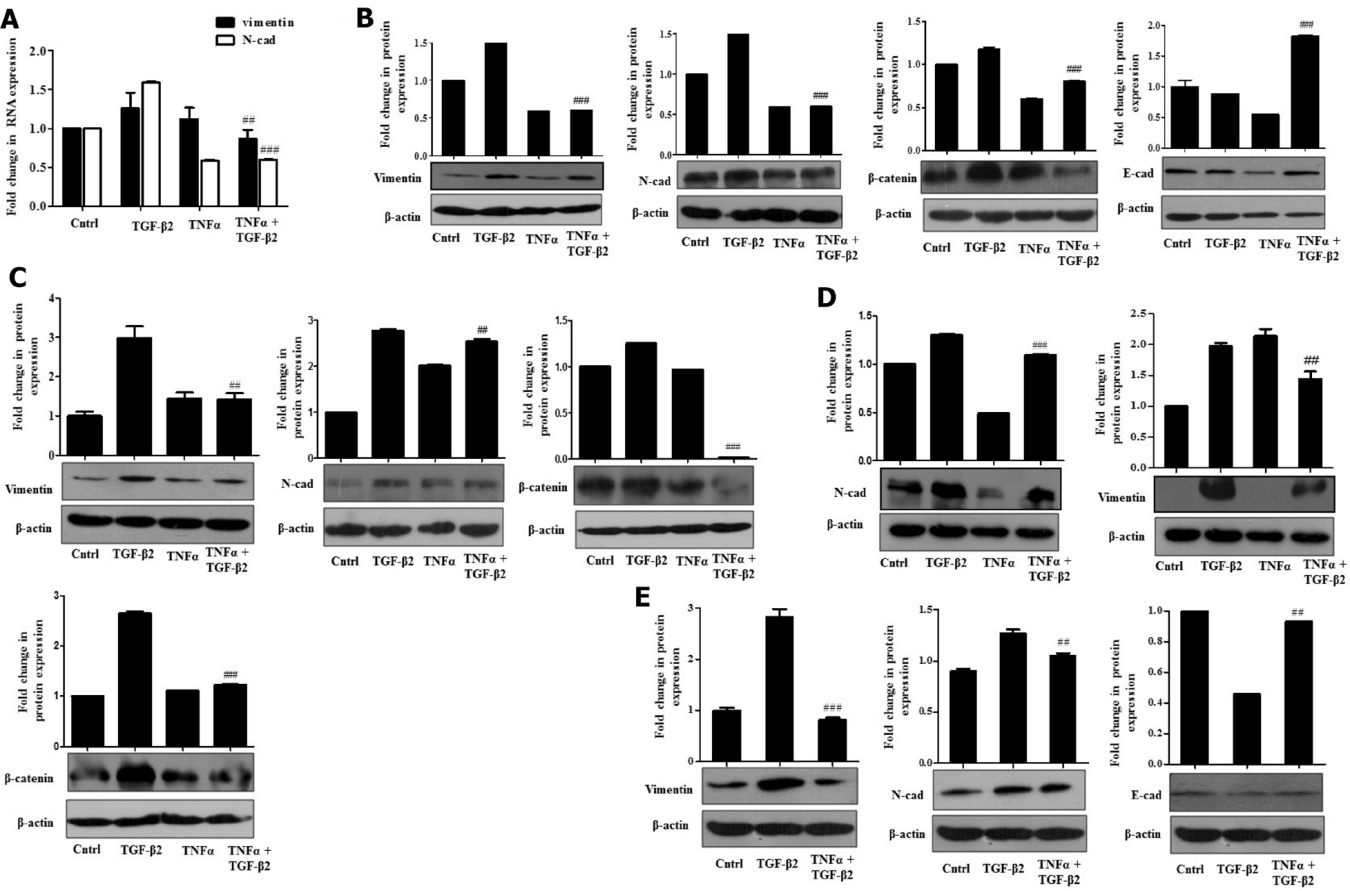
TNF-α suppresses TGF-β2-induced EMT (**A**) Real time PCR results showing expression of Vimentin and E-cadherin mRNA levels, upon simultaneous exposure of Huh7 cells to TGF-β2 (5 ng/ml) and TNF-α (20 ng/ml) for 48 h. (**B**) Immunoblot analysis showing protein expression of Vimentin, N-cadherin, β-catenin and E-cadherin upon simultaneous exposure of TGF-β2 (5 ng/ml) and TNF-α (20 ng/ml) for 48 h in Huh7 cells. (**C**) Immunoblot analysis showing protein expression of Vimentin, N-cadherin and β-catenin upon 72 h of simultaneous exposure to TGF-β2 (5 ng/ml) and TNF-α (20 ng/ml) in Huh7 cells. (**D**) Immunoblot analysis showing protein expression of N-cadherin and β-catenin upon 96 h of exposure to TGF-β2 (5 ng/ml) and TNF-α (20 ng/ml). (**E**) Immunoblot analysis showing protein expression of Vimentin, N-cadherin and E-cadherin upon exposure to TGF-β2 (5 ng/ml) and TNF-α (20 ng/ml) following rescue of serum with 10% FBS. Expression in untreated control was taken as arbitrary unit “1”. GAPDH expression was used as housekeeping control for RT-PCR and β-actin served as loading control for immunoblot.

Few studies have previously addressed the antagonistic activities exerted by pro-inflammatory cytokines and TGF-β. The opposing roles exerted by pro-inflammatory cytokines against TGF-β are known to play an essential role in context to maintaining tissue homeostasis and extracellular matrix deposition [28]. We therefore wondered whether the functionally opposing nature of these cytokines can represent a useful paradigm in the study of complex cellular signals regulating HCC pathogenesis. In this context, we explored the effect of TNF-α administration on TGF-β2–induced effects described above. TNF-α, generally released by activated macrophages, is a key player modulating inflammatory responses in the HCC tumor microenvironment [29]. Our findings reflect that Huh7 cells when exposed to TGF-β2 and TNF-α simultaneously, showed a significant reduction of TGF-β2-induced EMT features. A significant attenuation in mRNA expression of the EMT markers like, Vimentin and N-cadherin in TGF-β2 and TNF-α-treated samples was observed through RT-PCR (Figure 5A). Furthermore, immunoblot analysis also revealed a marked reduction in Vimentin, N-cadherin and β-catenin protein levels upon simultaneous exposure to TGF-β2 and TNF-α compared to only TGF-β2 treatment (Figure 5B). At the same time, E-cadherin expression substantially recovered in cells treated with TGF-β2 and TNF-α, when compared to only TGF-β2-treated cells (Figure 5B). Similar results were obtained at different time points studied where TNF-α antagonized TGF-β2 induced effects (Figure 5C and 5D). Immunoblot analysis showed a significant reduction in Vimentin and N-cadherin protein levels analyzed after 72 h and 96 h of TGF-β2 plus TNF-α exposure (Figure 5C and 5D). Moreover, a significant down-regulation of N-cadherin and Vimentin along with an up-regulation of E-cadherin in TGF-β2 and TNF-α-treated samples, was observed, even after serum rescue emphasizing the fact that TNF-α-mediated antagonistic effects are serum independent (Figure 5E). Similar results demonstrating an antagonistic effect of TNF-α over TGF-β2-mediated EMT were obtained in other epithelial cell types studied, like, lung cancer (H1299) and other HCC cell type (HepG2) suggesting a probable universal effect across different cell types (Supplementary Figure 5A and 5B).

### TNF-α inhibits TGF-β2–induced autophagy and signaling

**Figure 6:**
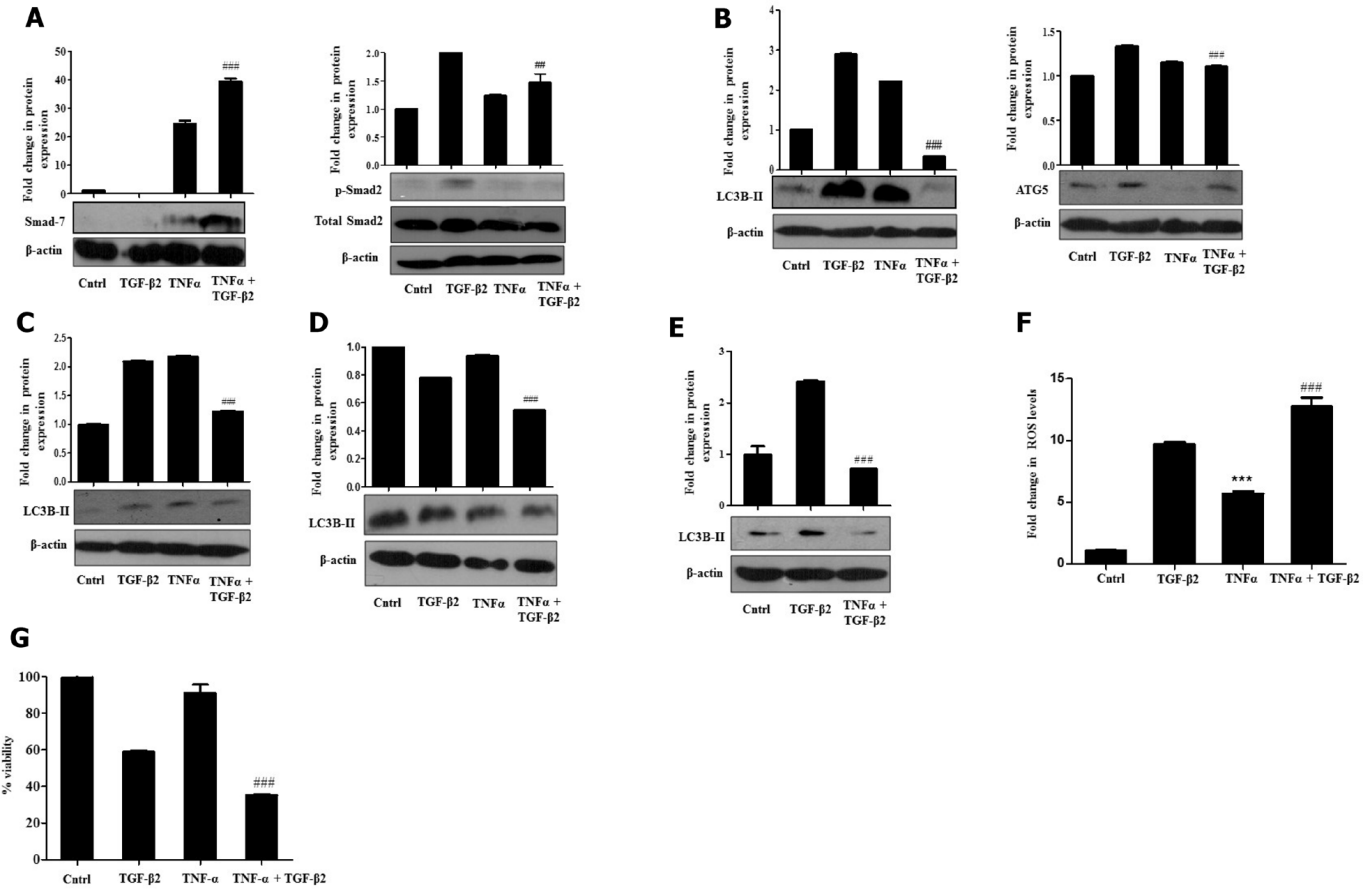
TNF-α antagonizes TGF-β2-induced signaling and autophagy (**A**) Immunoblot analysis showing smad7 and p-Smad-2 protein expression in Huh7 cells exposed to TNF-α (20 ng/ml, 48 h) along with TGF-β2 (5 ng/ml) for 48 h. (**B**) Immunoblot analysis of autophagic markers- LC3B-II and ATG5 in the presence or absence of TNF-α (20 ng/ml, 48 h) along with TGF-β2 (5 ng/ml). (**C**) Immunoblot analysis of LC3B-II post 72 h of TNF-α (20 ng/ml) and TGF-β2 (5 ng/ml) treatment. (**D**) Immunoblot analysis of LC3B-II post 96 h of TNF-α (20 ng/ml) and TGF-β2 (5 ng/ml) treatment. (**E**) Immunoblot analysis of LC3B-II upon simultaneous exposure to TGF-β2 (5 ng/ml) and TNF-α (20 ng/ml) for 48 h when serum was rescued with 10% FBS. (**F**) H2DCF-DA fluorimetric analysis showing elevated ROS levels upon simultaneous exposure to TGF-β2 (5 ng/ml) and TNF-α (20 ng/ml) for 48 h. (**G**) MTT assay showing percentage cell viability upon TGF-β2 (5 ng/ml) and TNF-α (20 ng/ml) treatment for 48 h, when compared to independent cytokine treatment or untreated cells.

More recently, existing studies have shown that several signaling pathways, activated in response to various stimuli, can lead to an increased expression of inhibitory Smad-7, which, in turn, prevents TGF-β-induced signaling [30]. Alternatively, specific cytokines have also been shown to directly interfere with Smad-2/3 functioning resulting in similar effects. Therefore, we checked for Smad-7 expression and activation of Smad-2, upon exposure to TGF-β2 and TNF-α. Interestingly, we observed a striking increase in Smad-7 levels and a stark decrease in phospho-protein levels of Smad-2 upon simultaneous exposure of both the cytokines (Figure 6A). Additionally, an attenuation in protein expression of TGF-β2-induced autophagic markers- LC3B-II and ATG5 was also observed in TGF-β2 and TNF-α-treated samples suggesting that a reduction of EMT like features could probably be attributed to inhibition of autophagy by TNF-α (Figure 6B). Similar results depicting a significant reduction of LC3B-II were obtained when cells were exposed to TGF-β2 for prolonged time periods like, 72 h and 96 h as well (Figure 6C and 6D). LC3B-II levels were found to go down significantly even after serum rescue suggesting TNF-α-mediated autophagy inhibition is a serum independent effect (Figure 6E). Interestingly, a significant elevation in ROS levels was also observed when Huh7 cells were exposed to both TGF-β2 and TNF-α (Figure 6F). To explore the effect of increased ROS on fate of cells, we analyzed viability of cells upon exposure to TGF-β2 and TNF-α. This resulted in an increased cytotoxicity of Huh7 cells when compared to any of the cytokine exposure independently (Figure 6G). Collectively our results suggest that TNF-α can abrogate TGF-β-induced metastatic spreading and proliferation of HCC cells by targeting autophagy signaling and regulating intra-cellular ROS levels.

### Transcriptomic analysis depicting TNF-α-mediated antagonism of TGF-β2-induced effects

**Figure 7:**
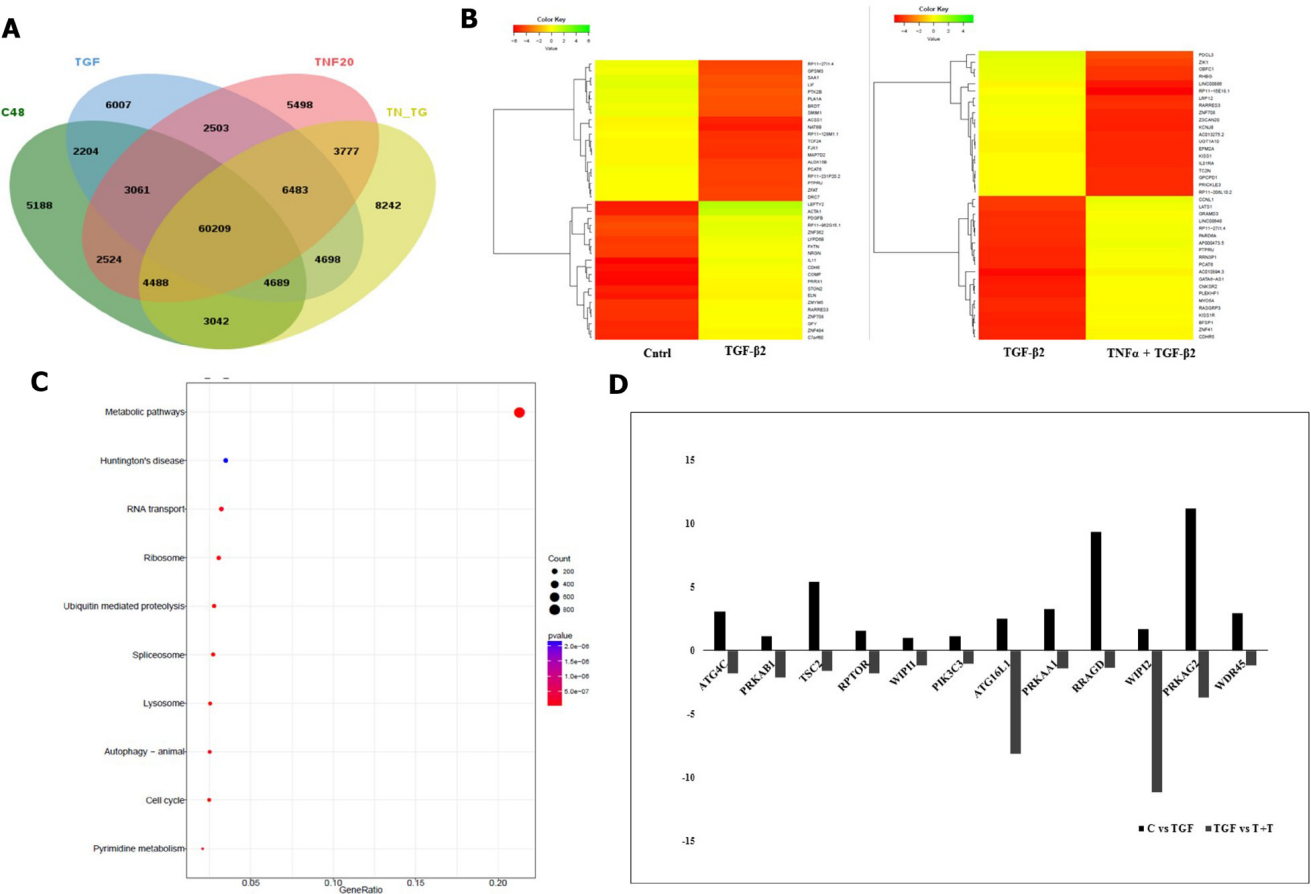
Transcriptomic analysis showing TNF-α-mediated antagonism of TGF-β2 induced effects (**A**) Venn diagram showing total number of transcripts and common transcripts across four samples sequenced- untreated control (C48), TGF-β2 (5 ng/ml; 48 h), TNF-α (20 ng/ml; 48 h) and TGF-β2 (5 ng/ml; 48 h) plus TNF-α (20 ng/ml; 48 h). (**B**) Heatmap demonstrating top 40 de-regulated transcripts in TGF-β2 (5 ng/ml; 48 h) treated samples when compared to untreated control; and in TGF-β2 (5 ng/ml; 48 h) plus TNF-α (20 ng/ml; 48 h) treated samples when compared to only TGF-β2 segregated based on their log2 fold change values. (**C**) Pathway enrichment analysis showing the top 10 pathways getting de-regulated post TGF-β2 treatment. (**D**) Graph showing up or down regulation of transcripts involved in autophagy, upon exposure to TGF-β2 (5 ng/ml; 48 h) in comparison to untreated control; and in TGF-β2 (5 ng/ml; 48 h) plus TNF-α (20 ng/ml; 48 h) treated samples when compared to only TGF-β2.

**Figure 8:**
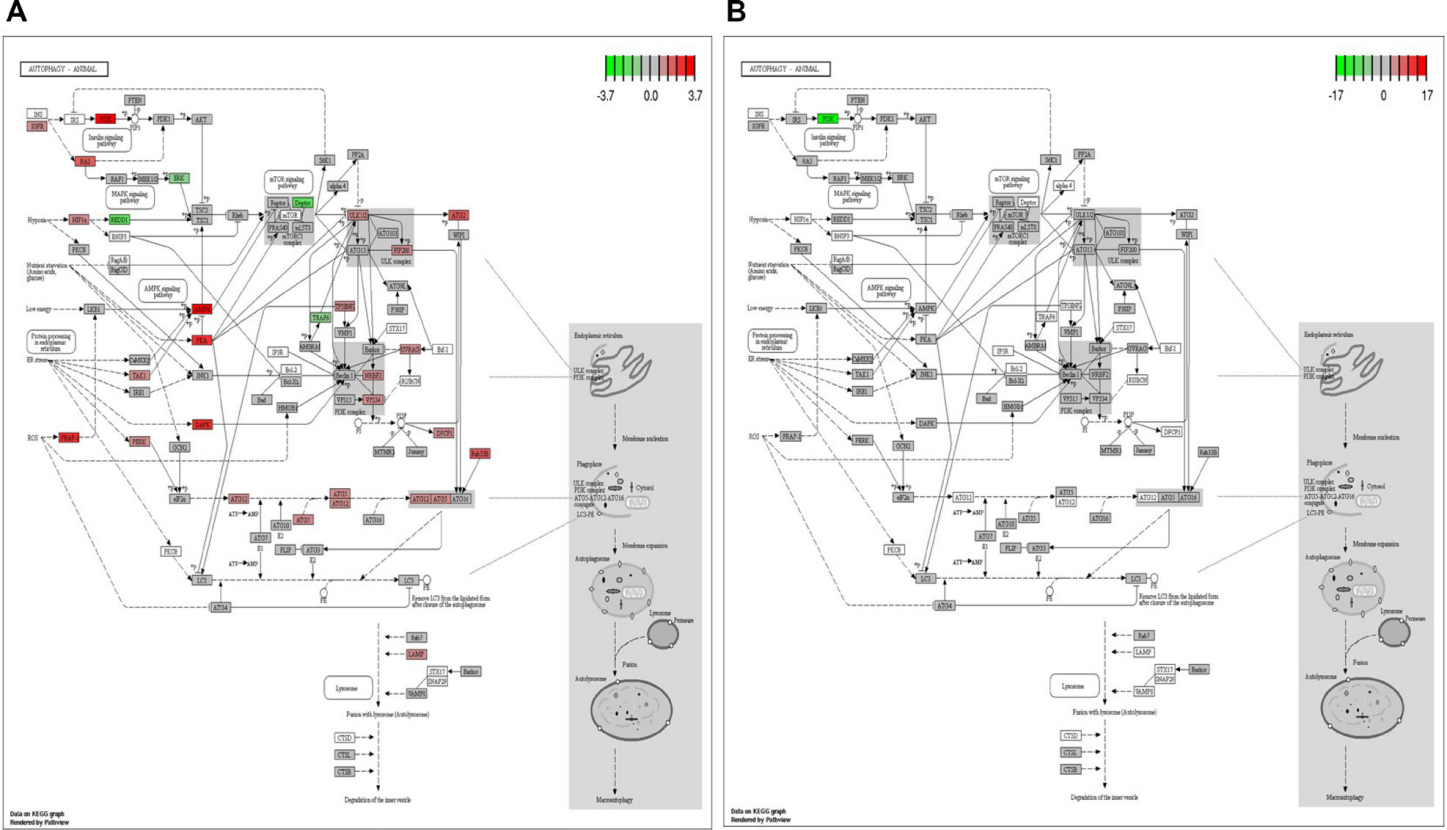
KEGG pathway graph rendered by Pathview KEGG Pathview showing autophagy pathway genes up or down regulated in TGF-β2 (5 ng/ml; 48 h) treated samples with respect to control (**A**), and in TGF-β2 (5 ng/ml; 48 h) plus TNF-α (20 ng/ml; 48 h) treated samples compared to only TGF-β2 (5 ng/ml; 48 h) (**B**).

Illumina paired end reads (150*2) were generated for untreated control, TGF-β2, TNF-α and TGF-β2 plus TNF-α exposed Huh7 cells and reference based transcriptome analysis was carried out. An average of 91.7% of the reads were aligned to the reference genome. Approximately, 89 K transcripts covering ∼22 K genes were found to be expressed across all the samples. On an average, approximately 32% of the transcripts were differentially regulated in cytokine-treated samples of which 6.2 K transcripts were found to be uniquely expressed and 6029 transcripts were found to be common across all the samples, which is represented in the form of a Venn diagram (Figure 7A). Top 40 de-regulated transcripts in TGF-β2 compared to control, and in TGF-β2 plus TNF-α compared to TGF-β2-exposed samples are represented in the form of a Heatmap (Figure 7B). The key genes regulating EMT, like, N-cadherin, Vimentin and β-catenin were found to be significantly up-regulated in TGF-β2-treated samples when compared to control with a log2fold change value of 2.28, 1.10281 and 1.15 respectively. Substantiating our previously observed results, the expression of N-cadherin, Vimentin and β-catenin were either neutrally regulated or significantly down-regulated in TGF-β2 plus TNF-α treated samples (N-cadherin-0.092569, Vim-0.174, β-catenin-0.98). Also, in corroboration to results described earlier, E-cadherin and Smad7 were found to be drastically going up with log2fold change values of 2.14 and 6.4 respectively following TGF-β2 plus TNF-α exposure in comparison to only TGF-β2-treated samples. To have a holistic idea on transcriptomic alterations associated with intracellular signaling, pathway enrichment analysis was performed. Amongst the top pathways that were severely de-regulated post exposure to cytokines, we observed that pathways related to autophagy signaling, lysosome and protein degradation machinery were the ones that were pre-dominantly altered (Figure 7C). This is suggestive of the fact that there is a significant alteration of intra-cellular material or protein turn-over post exposure to cytokines which might determine cellular fate. Importantly, in accordance to earlier results, a significant down-regulation of autophagic genes was observed in TGF-β2 plus TNF-α-treated samples when compared to only TGF-β2-exposed cells. The log2fold change in some of the key autophagy-related genes in response to cytokines is represented in the form of a graph (Figure 7D). Two key genes involved in autophagy which were found to be significantly down-regulated in TGF-β2 plus TNF-α-treated samples included, Atg16L1 and WIPI2. Atg16L1 along with other Atgs are some of the final Atgs recruited that determines the site of LC3-II formation; LC3 family of proteins are required for phagophore expansion, closure, and cargo recruitment. Whereas, WIPI2 acts immediately upstream of Atg16L1 and is responsible for Atg12-5-16L1 recruitment to omegasomes (autophagosomes arise from endoplasmic reticulum-derived omegasomes), resulting in LC3 lipidation and subsequent autophagy. Furthermore, KEGG autophagy pathway graph rendered by Pathview also shows an up-regulation of genes (in red) (Figure 8A) involved in autophagic pathway in TGF-β2-treated samples compared to control; however, the same genes did not show a significant up-regulation in TGF-β2 plus TNF-α-treated samples compared to only TGF-β2 (Figure 8B). Overall, the transcriptomic analysis authenticates and emphasizes on the up-regulation of autophagy supporting TGF-β2-induced EMT and attenuation of the same upon addition of TNF-α in Huh7 cells; we also provide valuable information on critical genes that are key to regulation of autophagy and EMT upon cytokine exposure.

## DISCUSSION

HCC is one of the most prevalent cancers worldwide with a relative 5-year survival rate as low as 15% [31]. This authenticates the need for more studies related to the understanding of the disease. The tumor microenvironment is known to play a critical role in the complex etiology of HCC where an intricate interplay exists between the hepatocytes and surrounding cells [32]. Cytokines like, TGF-β and TNF-α are secreted in the HCC tumor milieu, which alongside other cytokines, growth factors and tumor infiltrating leukocytes invariably create a chronic inflamed state contributing significantly to the progression of the disease [32]. Hence, in this study we investigated the effect of the above cytokines in HCC cells and their functional co-relation with respect to disease progression.

TGF-β, as a cytokine has a plethora of biological functions. TGF-β signaling is known to play a tumor suppressive function, attenuating cell growth and inducing apoptosis. On the contrary, TGF-β is one of the most powerful activator of EMT during tumor progression. TGF-β in late stages of cancers induces EMT, promoting cellular migration and anoikis resistance [33]. This complicates the biological understanding of the spectrum of its functional diversity encouraging more studies elucidating its role. Interestingly, autophagy seems to be amongst the wide plethora of cellular processes that is under the control of TGF-β. It is known to activate autophagy in both normal and cancer cells [9]. It has been observed that cancer cells exposed to TGF-β can result in a strong activation of autophagy marked by an increased expression of pro-autophagic genes. This effect is primarily facilitated by Smad signaling. Functional exploration of TGF-β-induced autophagy supports its role primarily in induction of apoptosis. Additional supportive evidences also emphasize on the fact that TGF-β-induced autophagy inhibits metastatic progression of cancer cells [34]. However, TGF-β can probably trigger both pro-tumorigenic and anti-tumorigenic signals and the choice may be completely dependent on the cellular context and the stage of tumor progression [33]. Interestingly, in our results, we observed that TGF-β2 treatment led to simultaneous induction of cytostasis and EMT like phenotype in Huh7 cells. The cells utilized autophagy as a pro-survival strategy, as inhibition of it by CQDP or siRNA-mediated ablation of ATG5, abrogated EMT-like features. Interestingly, Yang *et al.* in 2006 suggested that TGF-β, being a pleiotropic molecule can not only induce EMT, but can also simultaneously induce other cellular processes like, apoptosis in mouse hepatocyte cells [35]. This endorses the fact that TGF-β can concurrently regulate multiple biological functions. In corroboration to above, we observed that pro-survival autophagy was required for the Huh7 cells to promote EMT features. Our results are in corroboration to Pang *et al.* (2016) who showed that TGF-β can induce both autophagy and EMT in mouse tubular epithelial cells and inhibition of autophagy reduced TGF-β-induced EMT [36]. We speculate that, during metastatic spreading, a massive reorganization of cellular interaction properties and loss of the adhesion, can render cancer cells without an effective anchorage thus inducing apoptosis. Under these circumstances, autophagy can come to the rescue and induce resistance to cell death. In our study, pre-treatment of Huh7 cells with CQDP or siRNA against ATG5, resulted in down-regulation TGF-β-induced metastatic features, enhanced cytotoxicity and apoptotic cell death thus proving that autophagy was acting as a survival response facilitating EMT. Similar pro-survival role of autophagy has been observed before but it is completely context and cell type dependent [19].

**Figure 9:**
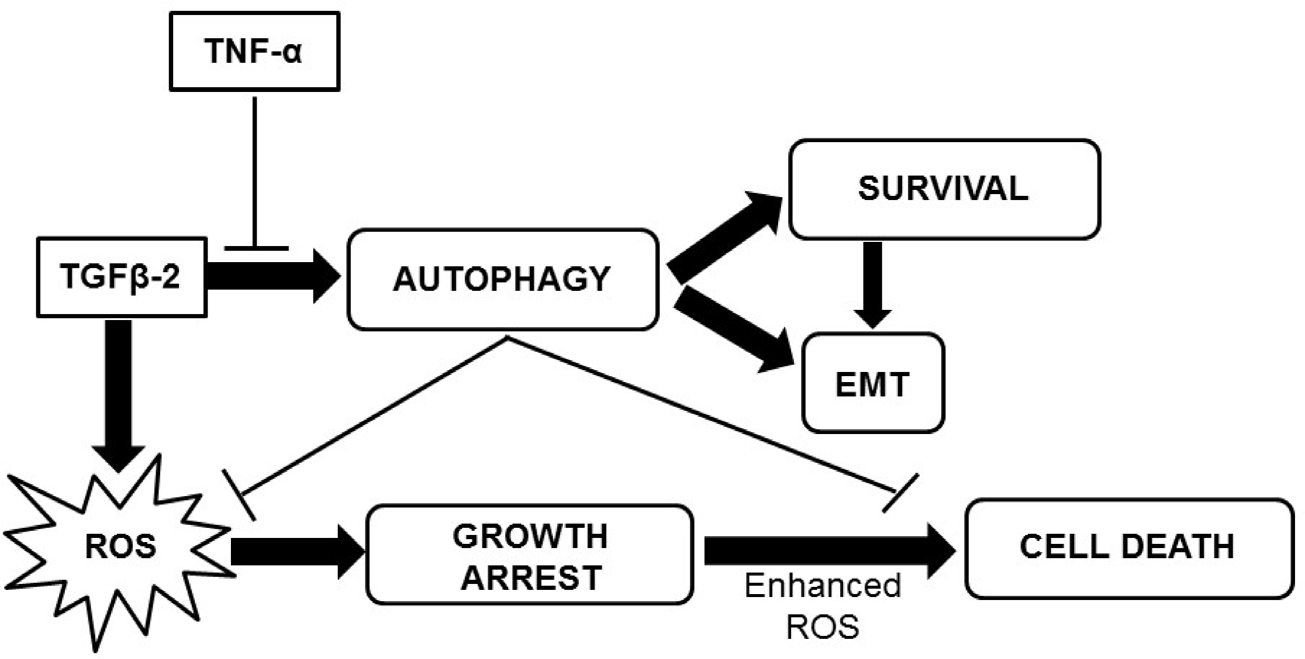
A schematic representation of the effect of TGF-β2 and TNF-α on Huh7 cells

Since, recent studies suggest that mitochondrial ROS are essential for TGF-β-mediated gene expression [37], we explored ROS levels upon TGF-β exposure as well. Significantly increased ROS levels were observed in cells exposed to TGF-β. Interestingly, quenching of ROS by NAC resulted in an increased expression of EMT markers. In contrary, inhibition of autophagy resulted in a significant enhancement in ROS levels suggesting that TGF-β-induced autophagy limits ROS facilitating EMT. We assume that this increase in ROS levels, upon autophagy inhibition, was the trigger to tip the balance of Huh7 cells from survival towards cell death. This role of ROS and autophagy in TGF-β-induced EMT is demonstrated through a schematic diagram (Figure 9).

Previous report suggests that the synthesis of type-I collagen is regulated by cytokines especially, TGF-β [38], and the matrix-remodeling function of the pro-inflammatory cytokine, TNF-α is opposite to TGF-β [39]. We hence explored the effect of TNF-α addition on TGF-β-induced effects in Huh7 cells. Interestingly, we observed that simultaneous exposure of TNF-α with TGF-β antagonized the function of later and attenuated TGF-β-induced Smad signaling and EMT. Similar results were obtained upon transcription profiling of TGF-β-treated samples and its comparison to TGF-β and TNF-α exposed cells. Apart from the EMT-specific genes like N-cadherin, Vimentin and others, which were significantly inhibited, two of the major autophagy genes which showed reduced expression on TNF-α exposure were Atg16L1 and WIPI2. These genes are required during autophagosome maturation and lipidation [40, 41], and with both the genes significantly going down in TGF-β2 plus TNF-α-treated samples we assume that TNF-α inhibits autophagy and hence EMT by suppressing the final steps of autophagosome maturation and lipidation.

Several pharmacological agents have till date been tested as a treatment modality to attenuate the effect of TGF-β, specifically, with respect to collagen deposition in fibrotic diseases. One approach has been to reduce TGF-β gene expression, either by suppressing transcription or by altering RNA stability; or by the use of antioxidants such as α-tocopherol [42]; or through administration of anti-TGF-β antiserum [43]. However, the use of antagonistically functioning cytokines to reduce TGF-β-induced effects in cancer cells is still lacking. In this regard, our study is unique and provides a novel and promising alternative for the treatment of HCC. Here, we provide concrete evidences that autophagy, a genetically regulated and finely orchestrated process of selective cell survival, induced by TGF-β2 can play a critical role in EMT sustenance of HCC cells and TNF-α antagonizes this effect by suppression of autophagy.

## MATERIALS AND METHODS

### Chemicals and reagents

TGF-β2 (#SRP3170), TNF-α (# H8916), 2′,7′-dichlorofluorescin diacetate (DCFDA, # D6883), monodansylcadaverine (MDC, # D4008), chloroquine di phosphate (CQDP, #C6528), propidium iodide (PI; #P4864) were purchased from Sigma; N-Acetyl-L-cysteine (NAC, #47866) and 3-(4,5-dimethylthiazol-2-yl)-2,5-di-phenyltetrazolium bromide (MTT, #33611) were obtained from SRL. FITC conjugated Annexin-V (#A13199), Annexin-binding buffer (#1796344) were procured from Thermo Fisher Scientific and JC-1 was bought from Santacruz (#sc-364116A). SIS3 was procured from (Bio vision # 2227-1), siATG5 was from Dharmacon # M-004374-04). Lipofectamine 3000 was from Invitrogen (#L3000-001). Unless otherwise mentioned, gene specific primary and secondary (goat anti-rabbit IgG) antibodies were obtained from Cell Signaling Technology (CST, USA).

### Cell culture

Human hepatocellular carcinoma cell line (Huh7) was cultured at 37°C, 5% CO_2_ in Dulbecco’s modified minimal essential medium (DMEM; Gibco, #12800-017) supplemented with 10% fetal bovine serum (FBS; Invitrogen, #26140-079) and 1% penicillin-streptomycin mixture (Invitrogen, #10378-016). Cells were grown till 50% confluency in DMEM with 10% FBS and rinsed with phosphate-buffered saline (PBS) and replaced with serum starved medium (DMEM containing 2% FBS) and kept for 12 h prior to cytokine treatments. For performing serum rescue experiment, 2 h post cytokine treatment, serum (10%) was added and cells were incubated with cytokine for 48 h.

### Cell viability assay

Assessment of cell viability was performed by MTT assay. Briefly, cells growing in log phase were cultured in 96-well plates overnight. The following day, cells were washed with PBS and starved for 12 h as mentioned above, post which cytokines at desired concentrations were added. Thereafter, MTT (20 µl; stock concentration- 5 mg/ml) was added. Cells were then incubated for 4 h; post which formazan crystals were solubilized in DMSO and readings were obtained at 495 nm with a differential filter at 630 nm using an enzyme-linked immune-sorbent assay (ELISA) micro-plate reader (Start-fax 2100). Percentage of viable cells were calculated using the formula: viability (%) = (mean absorbance value of drug-treated cells)/(mean absorbance value of control) *100 [44].

### RNA isolation and real-time PCR (RT-PCR)

Total RNA was isolated using TRIzol reagent (Sigma, #T9424). Complementary DNA (cDNA) was synthesized using GeneSure First Strand cDNA Synthesis kit (Genetix, # PGK162-B) with oligodT, as per manufacturer’s protocol. Templates were amplified using gene specific primers for Vimentin, N-cadherin and E-cadherin taking GAPDH as housekeeping control and detected using SYBR Green Supermix (Bio-Rad, #170-8882AP) in CFX Connect RT-PCR System (Bio-Rad). The primers used and their sequences are given below: - Vimentin- forward 5′- TCTACGAGGAGGAGATGCGG-3′, reverse 5′-GGTCAAGACGTGCCAGAGAC-3′; E-cadherin-forward 5′-TACACTGCCCAGGAGCCAGA-3′, reverse 5′-TGGCACCAGTGTCCGGATTA-3′; N-cadherin-forward 5′-CGAATGGATGAAAGACCCATCC-3′, reverse 5′-GGAGCCACTGCCTTCATAGTCAA-3′; GAPDH- forward 5′-GCACCGTCAAGGCTGAGAAC-3′, reverse 5′-TGGTGA AGACGCCAGTGG A-3′. The relative RNA expression was calculated using Livak method [45]. The melting temperature for the PCR reactions were 53°C for Vimentin and E-cadherin and 55°C for N-cadherin and the template was amplified for 30 cycles in each case.

### Immunoblotting

Immunoblotting was performed following protocols as described previously [46]. Cells were lysed in modified RIPA buffer (Sigma-Aldrich) and protein content was measured using Bradford reagent (Thermo Scientific). The cellular protein lysates were run in denaturing polyacrylamide gels and thereafter transferred to PVDF membrane (Thermo Fisher Scientific, #88518) for blocking with 5% skimmed milk (HiMedia). The blots were then probed or re-probed with specific primary antibodies and detected using enhanced chemi-luminescence (ECL; Thermo Fisher Scientific, #3210) detection system following the manufacturer’s protocol. The primary antibodies used were as follows: anti-E-cadherin (CST, 24E10), N-cadherin (CST, D4R1H), Vimentin (CST, D21H3), β-catenin (CST, D10A8), ATG5 (CST, D1G9), LC3-II (CST, D11), phospho-total Smad-2/3 (CST) and Smad-7 (Santacruz, #sc-365846). The secondary antibodies were horseradish peroxide-conjugated goat anti-rabbit IgG. Expression was quantitated using ImageJ and analyzed through Graph-pad Prism software [47].

### Determination of reactive oxygen species (ROS) levels

ROS levels were measured using 2, 7-dichlorofluorescein diacetate (H2DCF-DA) (Sigma) which measures intracellular generation of hydrogen peroxide, a procedure widely used for estimation of ROS. The H2DCF-DA passively enters the cell, where it reacts with ROS to form the highly fluorescent compound dichloro-fluorescein (DCF). Briefly, cells were seeded at a density of 6000 cells / well in 96 well plates, allowed to grow till 50% confluency. The ROS scavenger, N-acetyl cysteine (NAC, 5 mM) was added wherever mentioned, 1 h prior to treatment with cytokine to inhibit ROS. Following exposure, cells were washed with PBS and then incubated in 100 μl of working solution of H2DCF-DA (stock solution was diluted to yield a 10 μM working solution) at 37°C for 30 min. The fluorescence was measured at 485 nm excitation and 530 nm emission using a microplate reader (Fluoroskan Ascent) [48].

### Determination of mitochondrial membrane potential

Flow cytometric analysis of mitochondrial membrane potential was done using the cyanine dye JC-1 (5,5′,6,6′-tetrachloro-1,1′,3,3′-tetraethylbenzimi-dazolylcarbocyanine iodide). A shift in fluorescence intensity from red to green is an indication of mitochondrial depolarization. Huh7 cells were seeded at a density of 1 × 10^6^ cells/plate, grown overnight and then treated with specific cytokine alone or in combination with CQDP (added 1 h before cytokine addition). Cells were then harvested in 1ml of complete media. 5 μg/ml of JC-1 was added to cell suspension and vortexed vigorously. After incubating for 20 min at room temperature, samples were acquired using flow cytometer (Cytoflex, Beckmann Coulter) and analysis of acquired data was performed using CytExpert software [44].

### Analysis of autophagy using monodansylcadaverine (MDC)

The compound MDC, a specific autophago-lysosomal marker was used to analyze induction of autophagy [49–51]. For visualization of the autophagic vacuoles by fluorescence microscopy, cells were seeded on sterile cover slips and grown overnight. Following cytokine treatment, the cells were incubated for 10 min at 37°C with 0.05 mM MDC dissolved in PBS. The cover slips containing the cells were then washed with PBS and mounted with anti-fade mountant (containing DAPI). Intracellular MDC in the form of punctate dots were visualized using fluorescence microscopy. The images were taken at 100× under upright fluorescence microscope (Olympus, U-25ND25). For, fluorimetric measurement, cells after treatment were labeled with MDC for 10 min, washed with PBS and collected in 10 mM Tris-HCl (pH 8) containing 0.1% TritonX-100. Intracellular MDC was measured by fluorescence photometry (excitation 380 nm and emission 525 nm) in a microplate reader (Fluoroskan Ascent). An increase in MDC fluorescence upon treatment was expressed as fold change with respect to control.

### Flow cytometric analysis of cell cycle

For DNA content analysis, cells were seeded in 6 cm dishes at a density of 2 × 10^6^ cells/plate and grown overnight. After 48 h of treatment with cytokine, the cells were harvested, washed with PBS and centrifuged at 2000 rpm for 5 min at 4°C. The pellet was then re-suspended in 100 µL of PBS and 900 µL of ice cold 70% ethanol, used as a fixative. The fixed cells were incubated at 4°C overnight. The next day, cells were centrifuged and the pellet was re-suspended in 450 µL PBS with 10 µL of propidium iodide (PI; 2 mg/ml) containing solution [52]. The samples were then incubated in dark for 10 min followed by event acquisition using flow cytometer (Cytoflex, Beckmann Coulter) and analysis using CytExpert software.

### Flow cytometric detection of apoptosis

During apoptosis phosphatidylserine (PS) which under normal physiological conditions remain on the cytoplasmic surface of the lipid bi-layer gets exposed, which can be detected by fluorochrome-tagged protein AnnexinV that binds to phosphatidylserine residues in the presence of calcium ions. Hence, for determination of apoptosis, cells were seeded in 6 cm dishes at a density of 1 × 10^6^ cells/dish. The following day, the cells were treated with specific cytokines and incubated for 48 h. Thereafter, the cells were harvested, washed with PBS and re-suspended in 500 µL of 1× binding buffer (Thermo Fisher Scientific). To detect both early and late apoptotic cells, 4 µL of AnnexinV and 10 µl of PI were added to the cells in binding buffer, followed by incubation in dark for 20 min [52]. The samples were then acquired using flow cytometer (Cytoflex, Beckmann Coulter) and analysis of acquired data was performed using CytExpert software. Percentage of apoptotic cells was calculated by adding up percentage of cells in upper and lower right quadrant (UR and LR) is represented through bar diagram.

### ATG5 knockdown and SMAD inhibition studies

To inhibit macroautophagy, cells were stably transfected with siATG5 (a kind gift from Prof. Santosh Chauhan, ILS, Bhubaneswar) a crucial regulator of macro-autophagy using lipofectamine 3000. Studies were performed in Huh7 cells pre-incubated with 20 nM siATG5 for 6 hours followed by exposure to TGF-β2 for 48 h. For Smad inhibition, Huh7 cells were treated with 2.5 µM of SIS3 one hour prior to TGF-β2 exposure for 48 h.

### Transcriptomic analysis through RNA sequencing

Briefly, for transcriptomic sequencing total RNA was isolated and taken for fragmentation and priming. The fragmented and primed mRNA was further subjected to first strand synthesis followed by second strand synthesis. The double stranded cDNA was purified using HighPrep PCR magnetic beads (Magbio Genomics Inc, USA). The purified cDNA was end-repaired, adenylated and ligated to Illumina multiplex barcode adapters as per NEBNext^®^ Ultra™ Directional RNA Library Prep Kit protocol. The adapters used in the study were Illumina Universal Adapter: 5′-AATGATACGGCGACCACCGAGATCTACACTCTTTCCCTACACGACGCTCTTCCGATCT-3′ and Index Adapter: 5′-GATCGGAAGAGCACACGTCTGAACTCCAGTCAC ATCTCGTATGCCGTCTTCTGCTTG-3′. The adapter-ligated cDNA was purified using HighPrep beads and was subjected to Indexing-PCR to enrich. The final PCR product was purified with HighPrep beads. The sequencing library was initially quantified by Qubit fluorimeter (Thermo Fisher Scientific, USA) and its fragment size distribution was analyzed on Agilent Bio-analyzer. Finally, the sequencing library was quantified by qPCR using Kapa Library Quantification Kit (Kapa Biosystems, USA). The quantified libraries were pooled in equimolar amounts to create a final multiplexed library pool for sequencing on Illumina sequencer for 150bp paired-end reads. The cleaned reads were aligned to the reference genome using Tophat2. Transcript quantification was done using Cufflinks. Differential expression analysis was performed using Cuffdiff. Transcripts with log2fold change of 1 and above were considered as up regulated and those below -1 as down regulated. The raw reads were submitted to NCBI as BioProject PRJNA395629. Expression of some of the key genes regulating EMT was validated by RT-PCR, the conditions and primer sequences of which are described before.

### Statistical analysis

Tukey tests was used as a follow up to one way or two way ANOVA to compare every mean to a control mean and every mean with every other mean using Graph Pad Prism software version 5.0. The Bonferroni method was used to analyse multiple comparisons using Graph Pad Prism software version 5.0. The tests compute a confidence interval for the difference between the two means. Throughout the text the representative images are of experiments done in triplicates. Data represented in mean ± SEM (*n* = 6). The symbols in parenthesis denotes the following: ^*^p < 0.001 Vs Cntrl, ^#^*p* < 0.001 Vs TGF-β2, ns *P* > 0.05,^*^P ≤ 0.05, ^**^P ≤ 0.01, ^***^P ≤ 0.001.

## SUPPLEMENTARY MATERIALS FIGURES


