# Supplementary material for: TGF-β2-induced EMT is dampened by inhibition of autophagy and TNF-α treatment
Source: Oncotarget. 2018 Jan 4;9(5):6433–49. doi: 10.18632/oncotarget.23942 (PMC5814223; doi:10.18632/oncotarget.23942)
Supplement: Supplementary file 1 [file oncotarget-09-6433-s001.pdf]

TGF-β2-induced EMT is dampened by inhibition of autophagy and TNF-α treatment

SUPPLEMENTARY MATERIALS

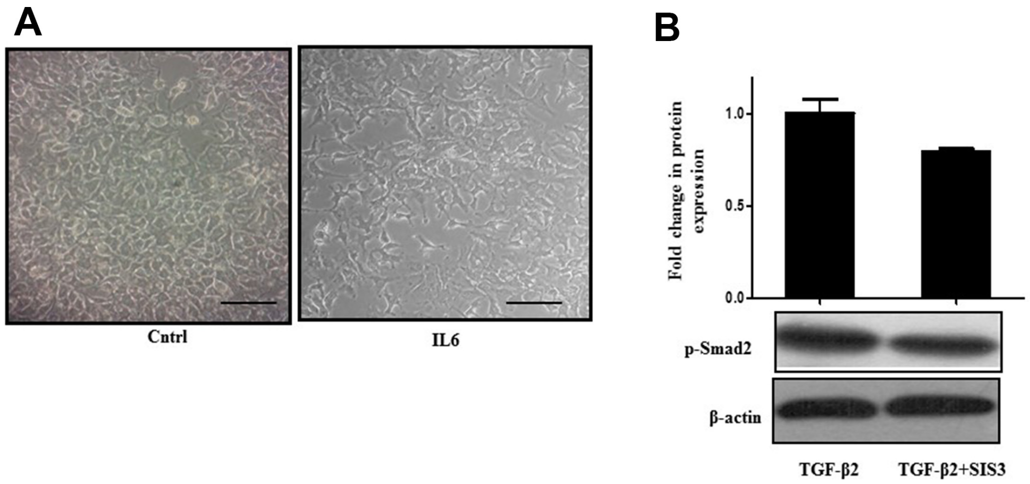

Supplementary Figure 1: (**A**) Phase contrast microscopic images showing morphology of Huh7 cells post 48 h of IL-6 treatment. (**B**) Immunoblot assay showing a down-regulation of p-Smad-2 after adding SIS3 (2.5 μM).

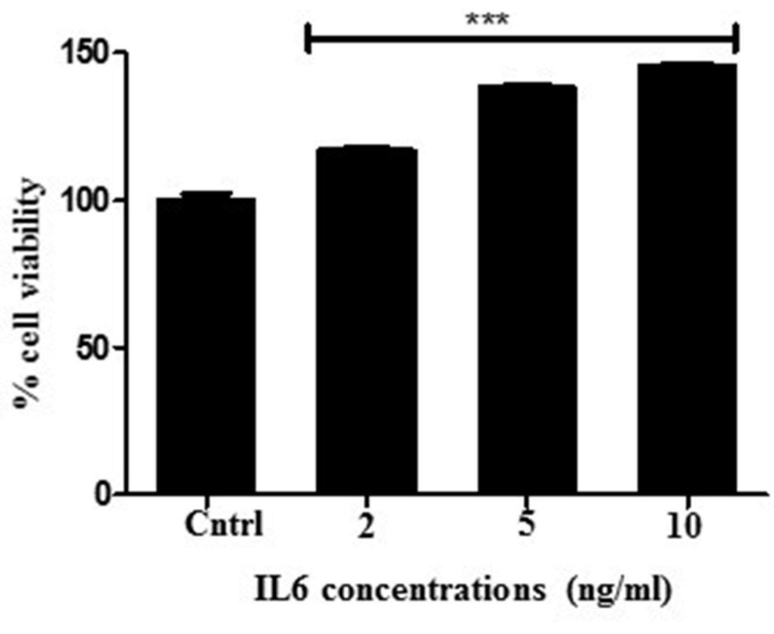

Supplementary Figure 2: Graph showing percentage viability of Huh7 cells measured through MTT assay upon exposure to varied doses of IL-6 for 48 h.

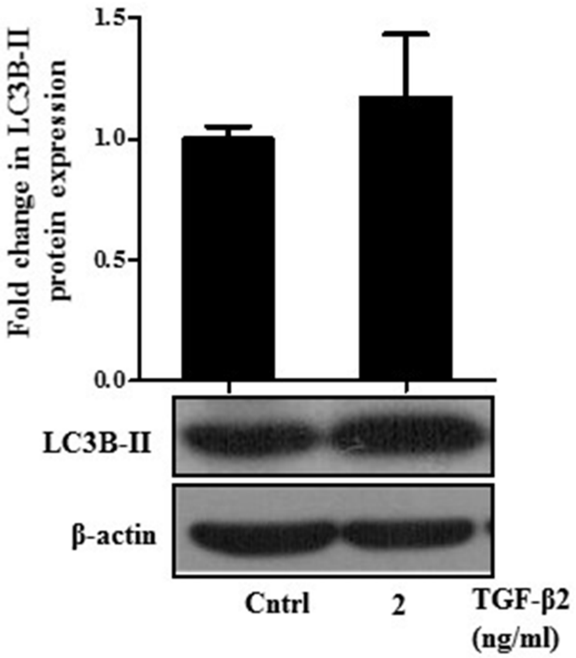

Supplementary Figure 3: Immunoblot assay showing up-regulation of LC3B-II after adding TGF-β2 (2 ng/ml for 48 h).

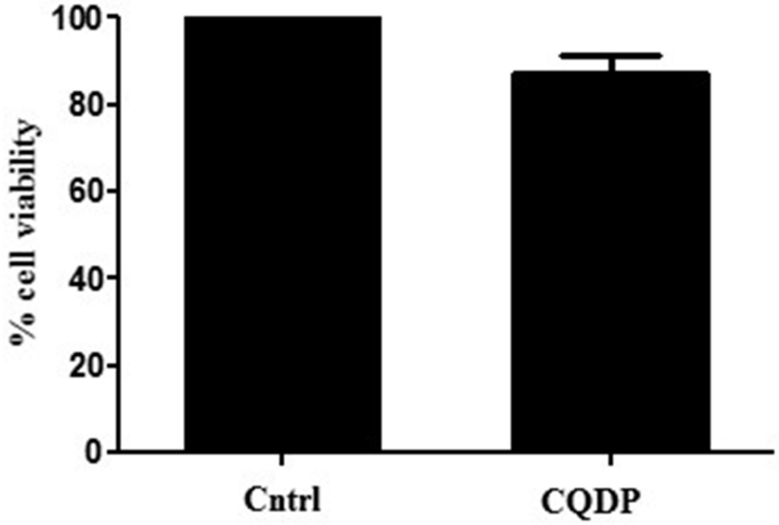

Supplementary Figure 4: Graph showing percentage viability of Huh7 cells measured through MTT assay upon exposure to CQDP for 48 h.

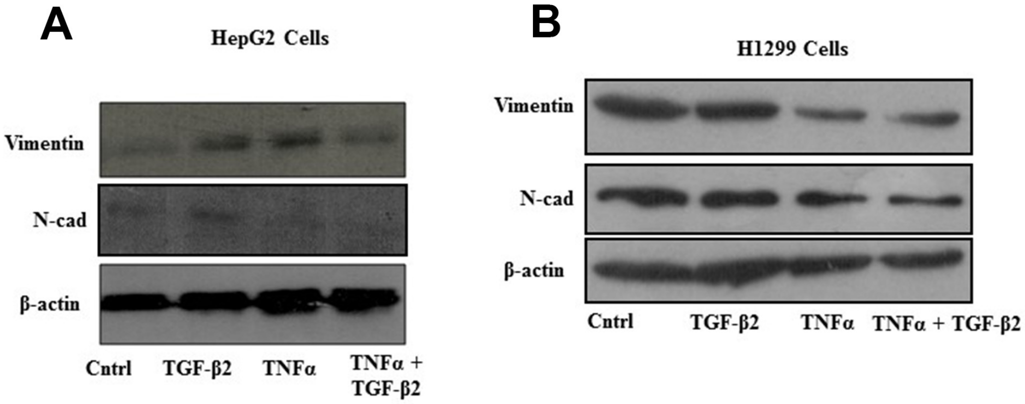

Supplementary Figure 5: (**A** and **B**) Immunoblot assay showing expression of EMT marker/s in HepG2 and H1299 cells after exposure to TGF-β2 (5 ng/ml) or TNF-α (20 ng/ml) or both for 48 h.
